# Prevalence and Risk Factors of Maternal Anxiety in Late Pregnancy in China

**DOI:** 10.3390/ijerph13050468

**Published:** 2016-05-04

**Authors:** Yu-ting Kang, Yan Yao, Jing Dou, Xin Guo, Shu-yue Li, Cai-ning Zhao, Hong-zhi Han, Bo Li

**Affiliations:** 1 School of Public Health, Jilin University, Changchun 130021, China; 18204319702@163.com (Y.-t.K.); zhaocn2711@mails.jlu.edu.cn (C.-n.Z.); 2 Department of Epidemiology and Biostatistics, School of Public Health, Jilin University, Changchun 130021, China; yaoyan@jlu.edu.cn (Y.Y.); 13526627874@163.com (J.D.); gggxxxfly@163.com (X.G.); 3 Center for Nursing Service, Changchun Maternity Hospital, Changchun 130021, China; kelly11637@sina.com; 4 Editorial Office of Journal of Jilin University, Medicine Edition, Changchun, Jilin 130012, China; hanhz@jlu.edu.cn

**Keywords:** antenatal anxiety, mental health, nursing, risk factors, cross-sectional study, chinese women

## Abstract

*Objective*: A large number of studies have shown the adverse neonatal outcomes of maternal psychological ill health. Given the potentially high prevalence of antenatal anxiety and few studies performed among Chinese people, the authors wanted to investigate the prevalence of antenatal anxiety and associated factors among pregnant women and to provide scientific basis to reduce prenatal anxiety effectively. *Methods*: A cross-sectional study was carried out at the Changchun Gynecology and Obstetrics Hospital from January 2015 to march 2015, with 467 participants of at least 38 weeks’ gestation enrolled. Antenatal anxiety was measured using the Self-Rating Anxiety Scale (SAS). χ^2^ test and logistic regression analysis were performed to evaluate the association of related factors of antenatal anxiety. *Results*: Among the 467 participants, the prevalence of antenatal anxiety was 20.6% (96 of 467). After adjustment for women’s socio-demographic characteristics (e.g., area, age, household income), multivariate logistical regression analysis revealed that antenatal anxiety showed significant relationship with education level lower than middle school (years ≤ 9), expected natural delivery, anemia during pregnancy, pregnancy-induced hypertension syndrome, disharmony in family relationship and life satisfaction. *Conclusions*: It is important to prevent or reduce antenatal anxiety from occurring by improving the health status of pregnant women and strengthening prenatal-related education and mental intervention.

## 1. Introduction

As the modern medical model develops, psychological health has been increasingly given more attention. Previous researches have shown a high prevalence of psychiatric illness in pregnant women [1,2], not only in the developed countries, but also in developing countries such as Turkey [3]. Furthermore, a large body of research exists on the adverse outcomes of maternal psychological ill health [4,5], most notably depression and anxiety during pregnancy. For example, psychiatric illness during pregnancy is considered to contribute to prematurity, low birth weight and obstetric complications [6,7].

Antenatal anxiety, as a common form of psychiatric illness, is a reflection of stress response [8], which occurs when personal well-being is threatened during pregnancy. Antenatal anxiety has been thought to be related to attention-deficit/hyperactivity disorder in children born to women who experience such antenatal anxiety [9,10,11,12]. It is believed that change in maternal hypothalamic–pituitary–adrenal (HPA) axis activity and fetal over exposure to glucocorticoids are potential pathogenetic factors in these adverse outcomes [13,14]. Findings from some studies indicated that anxiety is more common during pregnancy than depression and often co-morbid with depression, even related to the occurrence of postnatal depression across many countries [7,15,16]. Studies have examined the prevalence and its associated factors of postnatal depression [17], whereas only a few studies have explored the prevalence of anxiety in pregnancy.

Antenatal anxiety is believed to be a psycho-biological process, which means that it is also influenced by complex biological systems, particularly the endocrine system. The fluctuation of estrogen and progesterone may also induce anxiety among pregnant women [18]. Regarding the fact that endocrine system change largely, it is possible that antenatal anxiety rise as birth approaches [19].

In Chinese culture, there exists a widespread gender preference for male progeny. For a long time, this preference led to a high number of sex-selected abortions of female fetuses, contributing to an unequal male-to-female ratio. Today, fetal sex determination is forbidden, which may increase the level of anxiety in pregnant women who have a preference for a male child, because of the uncertainty of the fetal sex. A study in Bangladesh reported a 29% high prevalence of antenatal anxiety regarding the gender sensitivities in their country [20]. Similarly, there may be a potentially high prevalence of anxiety among Chinese women. While there are studies on postpartum depression and gender preference [21], little research is available on either the prevalence or factors associated with antenatal anxiety in China. A study in Hong Kong showed a negative relationship between feelings of control during labour and maternal anxiety. However, given the economic and cultural differences between Hong Kong and mainland China, it is difficult to extrapolate the findings of the study to our region of China [22]. Furthermore, the prevalence of antenatal anxiety varies widely from 9.1% to 59.5% assessed by different scales [19,23,24,25], scarce work has been done to examine the prevalence in China.

Thus, given the potential high prevalence and its possible adverse effects of antenatal anxiety [26], the authors wanted to determine the prevalence of prenatal anxiety in Changchun, Jilin Province, Northeast China, and to understand the associated risk factors during antepartum hospitalization. We hope that by raising the awareness of maternal prenatal anxiety among health care professionals and caregivers, mothers will be screened early in their pregnancy and offered personalized services for their psychological health in order to reduce, distress and improve pregnancy outcomes [27].

## 2. Experimental Section 

### 2.1. Study Design

A cross-sectional study was conducted at Changchun Maternity Hospital in the Jilin Province of China from January to March in 2015. Since we could not ensure random sampling due to lack of resources, we used a sampling group of hospitalized pregnant women awaiting delivery. The inclusion criteria included the following: Chinese nationality, a gestation of at least 38 weeks, a pregnancy without any complications, such as twins or known congenital anomalies, and the ability to complete the survey. Afterwards, face-to-face interviews with participants before delivery were carried out in private by trained research assistants using a structured questionnaire. After the participants were informed of the objectives of the study, those who were willing to participate in the survey signed written informed consent. Finally, we enrolled a sample group of 476 pregnant women who met the criteria for antenatal anxiety. We’ve gained the permission from the Ethics Committee of Public Health, Jilin University and were in accordance with the ethical standards (approval code: 2014-1128).

### 2.2. Data Collection

The potential risk factors of antenatal anxiety were selected, based on our literature search. 

#### 2.2.1. Demographics Questionnaire 

The demographics questionnaire was developed specially for the study. The following demographic variables were collected: age, height, pre-pregnancy and late pregnancy weight, household income per month, education level in three level bands, occupation, marital relationship, type of delivery, and information on current health status such as pregnancy-induced hypertension syndrome and prenatal anemia. Social supports and sleep quality were also obtained in the study.

#### 2.2.2. Age Groups

In China, most women marry and deliver a baby before they are 30 years of age. If a woman does not have a child after reaching 30 years of age, she and her partner will very likely experience pressure to do so from both parents and friends, so we have set 30 years of age as the limit to explore whether age can influence the anxiety level of pregnant women. Being ≥35 years old is believed to make a high risk pregnancy more likely, which can lead to pregnancy complications such as pregnancy-induced hypertension, and can increase the chances that the baby will suffer birth defects. According to the inclination of most Chinese women and based on clinical significance, we divided participants into three age groups.

#### 2.2.3. Personality 

The Big Five personality traits are strongly related to mental health. A study from Kaplan agreed that openness, agreeableness, and conscientiousness are intermingled [28], so we wanted to use more concise and representative personality traits in our paper. The personality subgroups in our study are ”extraversion”, ”neuroticism” and “agreeableness”.

#### 2.2.4. Morning Sickness 

The present study suggested that morning sickness and anxiety disorders are frequently observed among pregnant women [29]. Our hypothesis was that there is a potential association between psychiatric disorders and morning sickness. The recorded severity of the morning sickness in our study was based on self-reporting by the pregnant women. If a woman reported morning sickness that lasted for a long period of time and was more serious, she was placed into a “serious” subgroup. If a woman reported morning sickness that lasted for a shorter period of time and was less severe, she was placed into a “light” subgroup. The other women who reported morning sickness between “serious” and “light” were defined as “medium”.

#### 2.2.5. Postpartum Care and Premarital Examination 

In China, if pregnant women deliver babies, parents or maternity matrons usually take care of them and their babies. This phenomenon is called “postpartum care”. We think it is an important social support and also may be an important factor in antenatal anxiety. A premarital examination is the physical examination prior to marriage, which includes testing for inherited diseases and venereal diseases. Our hypothesis is that if a woman knows that she and her partner do not have any of those problems, she will be confident in regard to her own and her baby’s health. This examination may decrease the anxiety of pregnant women and may make them less likely to suffer antenatal anxiety.

#### 2.2.6. Antenatal Anxiety

The Self-Rating Anxiety Scale (SAS) developed to assess adult anxiety, has demonstrated adequate validity and acceptable reliability in a previous study [30]. The SAS includes 20 items using 4-point response options ranging from 1 (never) to 4 (very often) to capture symptoms of antenatal anxiety. The responses are used to give a total score that can range from 20 to 80 and then the standard score equal to the integral part of the figure of total score multiplied by 1.25. To identify women with antenatal anxiety, a standard score ≥50 has been recommended [31], with higher scores indicating higher levels of anxiety. To measure symptoms of antenatal anxiety in current study, the participants were required to answer the frequency of each items over the past week in SAS, which the authors believe to be a good reflection on their attitudes before delivery and can be deemed as a dependent variable. 

#### 2.2.7. Life Satisfaction

Life satisfaction was assessed by the Satisfaction With Life Scale (SWLS) in the study. The SWLS, developed originally in 1985, includes only five items; however, it has been proven to be valid and reliable in previous studies [32,33] and has been used widely across many countries, including China [34]. A seven-point response option ranging from 1 (strongly disagree) to 7 (strongly agree) was used to examine an individual’s satisfaction with their life. The responses were added together to give a total score, ranging from 5 to 35. On this scale, 20 scores represented the neutral attitude. Scores between 5 and 9 represented extreme dissatisfaction with life, scores between 15 and 19 represented slight dissatisfaction with life, scores between 21 and 25 represented slight satisfaction with life, and scores ranging from 31 to 35 represented extreme satisfaction with life [35]. However, in our study, we combined groups representing extreme dissatisfaction, dissatisfaction, slight dissatisfaction, neutrality, and slight satisfaction; that is, scores ranging from 5 to 25 were considered to be dissatisfied with life. After transition, life satisfaction was seen as independent for analysis.

### 2.3. Data Analysis 

Data were entered into IBM SPSS statistical software program, version 19.0 (International Business Machines Corp., Armonk, NY, USA), and were then analyzed after being checked and corrected for any errors, including continuous variables (age, anxiety score, life satisfaction score) and categorical variables (area, income level, education level, occupation, personal character, experience of support from the partner and family, expected birth mode, postpartum care, premarital examination, anemia before or during pregnancy, gestational diabetes, calf cramps, pregnancy-induced hypertension, sleep deprivation, morning sickness). Based on Chinese cultural attitudes and the available literature [21,36], we converted continuous variables into categorical variables to achieve better analysis (details Table 1). Frequency distributions and descriptive statistics for major demographic variables were computed. The χ^2^ test was used to analyze the significant associations between variables and antenatal anxiety. Logistic regression is used to identify significant variables by removing confounding factors. We posit that it is more accurate and acceptable to include those potentially revealing variates (*p* ≤ 0.20) for use in adjustments [37]. Therefore, the variables were included in the bivariate logistic regression model to determine their independent contribution to antenatal anxiety and whether they had a significant association (*p* ≤ 0.20) according to the χ^2^ test or whether they were deemed important clinically even without statistical significance, as would be true in the case of gestational diabetes mellitus, for example. To estimate the strength of associations, odds ratios (*OR*) and 95% confidence intervals (*CI*) were presented for all variables in bivariate regression models. An α level of 0.05 was used for statistical tests.

## 3. Results

### 3.1. Participants

Because we conducted face-to-face interviews in our study, trained nurses collected the questionnaires after completing the interviews, so there are no missing data. The mean age was 28.9 years (standard deviation (*SD*) = 4.3). In our study, the majority of pregnant women reported high-school or technical secondary school degree (48.2%) followed by college or above degree (37.3%) and middle school or below degree (14.1%). Occupations involving mental labour (45.0%), including administration, medicine, education, and recreation and sports were most common, followed by physical labour (43.6%), including agriculture and livestock, service industry, and others (11.4%), which included occupations that could not be classified such as housewife, massage therapist, *etc.* Most women (54.0%) were from urban areas; 29.6% came from the surrounding county, and 16.5% were from rural areas. Furthermore, 44.1% of average household income per month was below 3000 Chinese yuan ($471), which is the average household income per month in Jilin, China, according to National Bureau of Statistics of the People’s Republic of China.

### 3.2. Prevalence of Antenatal Anxiety 

Slight and moderate anxiety was reported by 18.4% and 2.1% women respectively giving an overall prevalence of antenatal anxiety of 20.6% (96 of 467; 95% CI 17.0%–24.5%). No severe antenatal anxiety was found in the study.

### 3.3. Univariate Analysis of Influencing Factors for Antenatal Anxiety

We examined relationships between demographic characteristics and antenatal anxiety. Significant differences in the prevalence of antenatal anxiety were found between groups with different characteristics, such as educational level, conjugal and family relationships, expected birth mode, pregnant-induced hypertension, sleep deprivation, and life satisfaction (Table 1). The prevalence of antenatal anxiety was higher among women with middle school or below degrees, women with disharmony in conjugal or family relationship. Furthermore, antenatal anxiety was more common among women expecting to give birth through natural delivery than those anticipating caesarean sections. Women with pregnancy-induced hypertension or lower life satisfaction had higher prevalence of antenatal anxiety.

### 3.4. Multivariate Analysis of Influencing Factors for Antenatal Anxiety by Logistic Regression Model

Variables were included into the bivariate logistic regression model if they had significant association (*p* ≤ 0.20) by the χ^2^ test or deemed important enough clinically despite no statistical significance. These variables included area, educational level, conjugal relationship, family relationship, expected birth mode, anemia before pregnancy, anemia during pregnancy, pregnancy-induced hypertension, sleep deprivation, morning sickness, and life satisfaction. After adjustment for socio-demographic characteristics (e.g., area, age, household income), the results of logistic regression analysis showed that antenatal anxiety was predicted significantly by lower education level, expected natural delivery, anemia during pregnancy, pregnancy-induced hypertension syndrome, and disharmony family relationship (Table 2).

## 4. Discussion

Although obstetric intervention for physical care of pregnant women has improved dramatically in China over the past several decades, little attention has been paid to emotional care. This study is performed to assess the incidence of antenatal anxiety during pregnancy as well as associated risk factors in the Chinese population in mainland China. 

This study finds that antenatal anxiety is prevalent in approximately one-fifth of the pregnant women in this study (20.6%), which is consistent with a Brazilian study that found a high prevalence of antenatal anxiety [38]. In contrast, studies from developed Asian countries have reported a lower prevalence of such anxieties. For instance, a study on a sample of Singaporean women who were hospitalized during pregnancy shows that 12.5% of those women suffered from anxiety disorders [39]. Although socio-economic factors can cause anxiety, pregnancy could be an important alternative explanation. Most of the participants surveyed in our study were at least 38 weeks into their pregnancy, which is a point when they could deliver at any time. Therefore, it is likely that most of these women, who were in their third trimester, were experiencing a marked amount of physical discomfort, which could lead to anxiety [40]. Although the prevalence of estimated antenatal anxiety may vary significantly across different studies due to different sampling methodologies and measurement errors [41,42], these studies agree that anxiety is a common and significant problem during pregnancy and that antenatal anxiety has become an important public health issue, particularly in developing countries.

Moreover, our study shows that pregnancy-induced hypertension syndrome and anemia during pregnancy are the major risk factors of antenatal anxiety among pregnant women. However, to our knowledge, the literature on antenatal anxiety with pregnancy complication is limited. A previous research had shown that pregnant women in hospitals tended to become more anxious and vulnerable when they were in poor health [23], which is consistent with our study. Also, in another study, pregnant women with preeclampsia reported complete shock and tended to suffer from high anxiety due to fear of babies’ prematurity, loss and guilt [43]. Therefore, pregnant women with complications should be given more psychological care from caregivers and their families, which could diminish the occurrence and development of antenatal anxiety.

Besides, this study has revealed that anxiety during pregnancy is associated with natural delivery method. To our surprise, our study found that planned cesarean deliveries, including those requested by the mother without medical reasons, were more common than planned natural deliveries (283 *vs.* 184). The fear of giving birth and experiencing a natural delivery is a real challenge for women, and this fear is strongly linked to the request for a cesarean section. Pang *et al.* found that women voluntarily elected cesarean sections after having their first child, even when they had attempted to give birth naturally in the past [37]. There are many unknown factors during pregnancy that cannot be controlled and that sometimes result in emergency cesarean deliveries, including situations involving intrauterine growth restriction, which eventually threatens the mother’s or the baby’s life. In addition, pregnant women are conscious during natural childbirth, are without the use of analgesics or anesthetics, and experience labor pain for at least a few hours. We suggest that these worries and fears contribute to the escalating prevalence of antenatal anxiety. According to Maier, for pregnant women, a planned cesarean section is a well-tolerated procedure, psychologically, when compared to natural childbirth [44]. Thus, as psychophysical care is an integral part of childbirth, professionals and caregivers need to pay close attention to women’s psychological status in the management of pregnancy and labor pain.

Our study finds a statistically significant relation between disharmony in family relationship and anxiety, whereas, conjugal relationship is believed to be important for mental health disorders. However, the exact mechanism by which disharmony in family relationship affects anxiety remains unclear. We infer that a lack of social support is a possible factor that eventually influences help-seeking behaviors [45]. Some studies have found that people in lack of social support tend to suffer from mental illnesses than those with adequate social support. Pregnant women in absence of social support are apt to be pessimistic and suffer from low self-esteem or self-worth [46]. Interestingly, this is consistent with studies on paternal anxiety during pregnancy, which is due to low self-esteem and poor social support [47]. In particular, individuals in these surroundings of disharmony are less likely to seek useful support or help, suggesting the importance of social support during pregnancy, especially family care.

In our study, we also found that pregnant women with lower levels of education were at a higher risk for developing anxiety during pregnancy. Some other studies have also found this association between poor education and mental health [48]. A lower level of education is correlated with a lower socioeconomic status, and individuals with these qualities lack adequate resources and information to improve their situation during pregnancy. A job-education mismatch is believed to influence mental health [49]. In our study, although there was not a significant relationship between the participants’ type of job and their anxiety level, we think education may have a stronger contribution to antenatal anxiety. Further, the results of our study indicate a significant association between life satisfaction and antenatal anxiety. In this respect, our findings highlight the importance of screening pregnant women dissatisfied with their lives so that the professional caregivers can provide more psychological care to the most vulnerable ones. In this way, we can recognize and decrease the incidence and the harmful consequences of antenatal anxiety effectively. 

## 5. Limitations

Our study has the following limitations: First, because of limited resources and time constraints, we conducted the study in only one regional hospital in Changchun where the available sample group was small; thus, the results are not representative all of pregnant women in China. Secondly, gender preference, an important factor in the psychological health of pregnant Chinese women, and its influence on antenatal anxiety, could not be assessed in our study. Third, as a cross-sectional design study, we could not identify the change of anxiety throughout pregnancy, and finally, this study was not designed to study the relationship between anxiety and depression during pregnancy, nor its impact on psychological health in postpartum period. 

## 6. Conclusions

Our study has shown that pregnancy-induced hypertension syndrome, anemia during pregnancy, lower degree of education, expected natural delivery, and life dissatisfaction are the main risk factors of antenatal anxiety among pregnant women. Thus, it is important to identify women at risk and to reduce antenatal anxiety occurring by improving the health status of pregnant women and strengthening prenatal related education and mental health intervention.

## Figures and Tables

**Table 1:** Socio-demographic characteristics and health states of the total participants (*n* = 467).

Characteristics	No Antenatal Anxiety *n* = 371	Antenatal Anxiety *n* = 96	χ^2^	*p* Value
	n (%)	n (%)		
Area				
Urban	203 (54.7)	49 (51.0)	3.723	0.155
County	113 (30.5)	25 (26.0)		
Rural	55 (14.8)	22 (22.9)		
Age (years)				
<30	240 (64.7)	69 (71.9)	2.571	0.277
30–35	95 (25.6)	22 (22.9)		
≥35	36 (9.7)	5 (5.2)		
Household Income (￥)				
<3000	167 (45.0)	39 (40.6)	0.600	0.440
≥3000	204 (55.0)	57 (59.4)		
Educational Level				
Middle School or Below	42 (11.3)	24 (25.0)	13.705	0.001
High School or Technical Secondary School	179 (48.2)	46 (47.9)		
College or Higher	150 (40.4)	26 (27.1)		
Job				
Muscular Work	172 (46.4)	43 (46.9)	0.767	0.681
Mental Work	159 (42.9)	45 (46.9)		
Other ^a^	40 (10.8)	8 (8.3)		
Personality				
Extraversion	167 (45.0)	40 (41.7)	1.250	0.535
Neuroticism	70 (18.9)	23 (24.0)		
Agreeableness	134 (36.1)	33 (34.4)		
Conjugal Relationship				
Harmony	330 (88.9)	64 (66.7)	28.712	<0.001
Disharmony	41 (11.1)	32 (33.3)		
Family Relationship				
Harmony	329 (88.7)	60 (62.5)	37.569	<0.001
Disharmony	42 (11.3)	36 (37.5)		
Expected Birth Mode				
Natural Delivery	136 (36.7)	48 (50.0)	5.686	0.017
Caesarean Sections	235 (63.3)	48 (50.0)		
Postpartum Care				
Yes	281 (75.7)	68 (70.8)	0.973	0.324
No	90 (24.3)	28 (29.2)		
Premarital Examination				
Yes	207 (55.8)	50 (52.1)	0.425	0.515
No	164 (44.2)	46 (47.9)		
Anemia Before Pregnancy				
Yes	17 (4.6)	8 (8.3)	2.118	0.146
No	354 (95.4)	88 (91.7)		
Anemia During Pregnancy				
Yes	329 (88.7)	42 (11.3)	2.819	0.093
No	79 (82.3)	17 (17.7)		
Gestational Diabetes Mellitus				
Yes	17 (4.6)	7 (7.3)	0.660	0.417
No	354 (95.4)	89(92.7)		
Calf Cramps				
Yes	97 (26.1)	30 (31.3)	1.004	0.316
No	274 (73.9)	66 (68.8)		
Pregnancy-Induced Hypertension				
Yes	12 (3.2)	13 (13.5)	15.991	<0.001
No	359 (96.8)	83 (86.5)		
Sleep Deprivation				
Worse	40 (10.8)	5 (5.2)	6.194	0.045
The Same	263 (70.9)	80 (83.3)		
Better	68 (18.3)	11 (11.5)		
Morning Sickness				
Serious	54 (14.6)	22 (22.9)	5.543	0.063
Medium	229 (61.7)	59 (61.5)		
Light	88 (23.7)	16 (15.6)		
Life Satisfaction				
Yes	340 (91.6)	58 (60.4)	59.062	<0.001
No	31 (8.4)	38 (39.6)		

^a^ Refers to occupations that could not be classified such as, housewife, massage therapist, *etc.*

**Table 2:** Logistic regression analysis for variables predicting antenatal anxiety symptoms.

Variables	β	Wald	*p* Value	OR (95% CI)
College or Higher ^a^	−1.052	7.267	0.007	0.349 (0.162–0.750)
Harmony in the Family Relationship ^b^	−0.939	8.913	0.003	0.391 (0.211–0.724)
Anemia During Pregnancy	0.870	5.686	0.017	2.387 (1.17–4.878)
Pregnancy-Induced Hypertension	1.818	14.667	<0.001	6.173 (2.427–15.625)
Expected Caesarean Sections ^c^	−0.799	8.802	0.003	0.450 (0.265–0.762)
Life Satisfaction	−1.744	29.617	<0.001	0.175 (0.093–0.328)

Results of multiple logistic regression analysis. Antenatal anxiety was the dependent variable; The selected factors were the independent variables. β standardized partial regression coefficient. ^a^ Middle school or below education level as reference or comparison group.^b^ Family relationship disharmony as reference or comparison group.^c^ Expected natural delivery as reference or comparison group.

## Abbreviations

The following abbreviations are used in this manuscript:

SAS: the Self-Rating Anxiety Scale
HPA: hypothalamic-pituitary–adrenal
SWLS: the Satisfaction With Life Scale
